# Hypovascular aggressive angiomyxoma in a pregnant adolescent: A case report and review of its multimodal imaging appearances

**DOI:** 10.1016/j.radcr.2026.03.007

**Published:** 2026-04-08

**Authors:** Kyler Shin, Kweku Agyepong, Craig Kym, Mohammad Ghasemi-Rad, David Léon

**Affiliations:** Department of Radiology, Baylor College of Medicine, 1 Baylor Plaza, Houston, TX

**Keywords:** Aggressive angiomyxoma, Hypovascular, Pregnancy, Leuprolide, Targeted embolization, Rectovaginal fistula

## Abstract

Aggressive angiomyxoma (AAM) is a rare mesenchymal tumor that primarily arises in the perineum and pelvis of young females. It is sometimes identified incidentally on pelvic examination and obstetric imaging. Although asymptomatic at small sizes, AAM is typically hypervascular and can grow large enough to complicate a patient’s obstetric and gynecologic course of treatment. While AAM is a rare diagnosis reported in the literature, there are limited descriptions of its multimodal radiologic appearance, and uncommonly, it can present as a hypovascular mass. We describe a rare case of hypovascular AAM in pregnancy that did not respond favorably to targeted embolization, with a special focus on its multimodal imaging timeline.

## Introduction

Aggressive angiomyxoma (AAM) is an uncommon, indolent, low-grade neoplasm of vulvo-perineal origin, usually in reproductive-age women. It tends to invade locally and can rarely metastasize to the lungs, peritoneum, and lymph nodes [1]. The standard of care is complete resection, although the current literature shows that up to 40% of cases exhibit recurrence.

AAMs commonly have estrogen and progesterone receptors, which enable them to grow in hormone-dependent states like pregnancy and respond to antagonistic hormonal therapy like leuprolide [1,2]. Hypervascularity is another feature that promotes growth in most AAMs, which often respond to targeted embolization [2]. If identified late or left untreated, these tumors can grow extremely large, thereby complicating the management options available during the antepartum period. Medical imaging plays a crucial role in identifying these masses and describing how they interact with surrounding structures, which can determine resectability or, in the case of pregnancy, vaginal versus cesarean delivery. Although a few cases of AAM in pregnancy have been reported throughout the literature, it is especially unusual for AAM to present as a hypovascular mass on contrast-enhanced imaging, with subsequent poor response to targeted embolization.

## Case report

A 16-year-old G1P0 patient at 22 weeks gestational age with a history of irregular menses presented to the hospital from an ultrasound clinic for additional imaging of an enlarging rectovaginal mass discovered on physical exam during her first prenatal visit at 17 weeks. The patient had been asymptomatic except for a small amount of blood when wiping. There was no history of constipation, difficulty with urination, abdominal pain, or pressure.

**Fig. 1:**
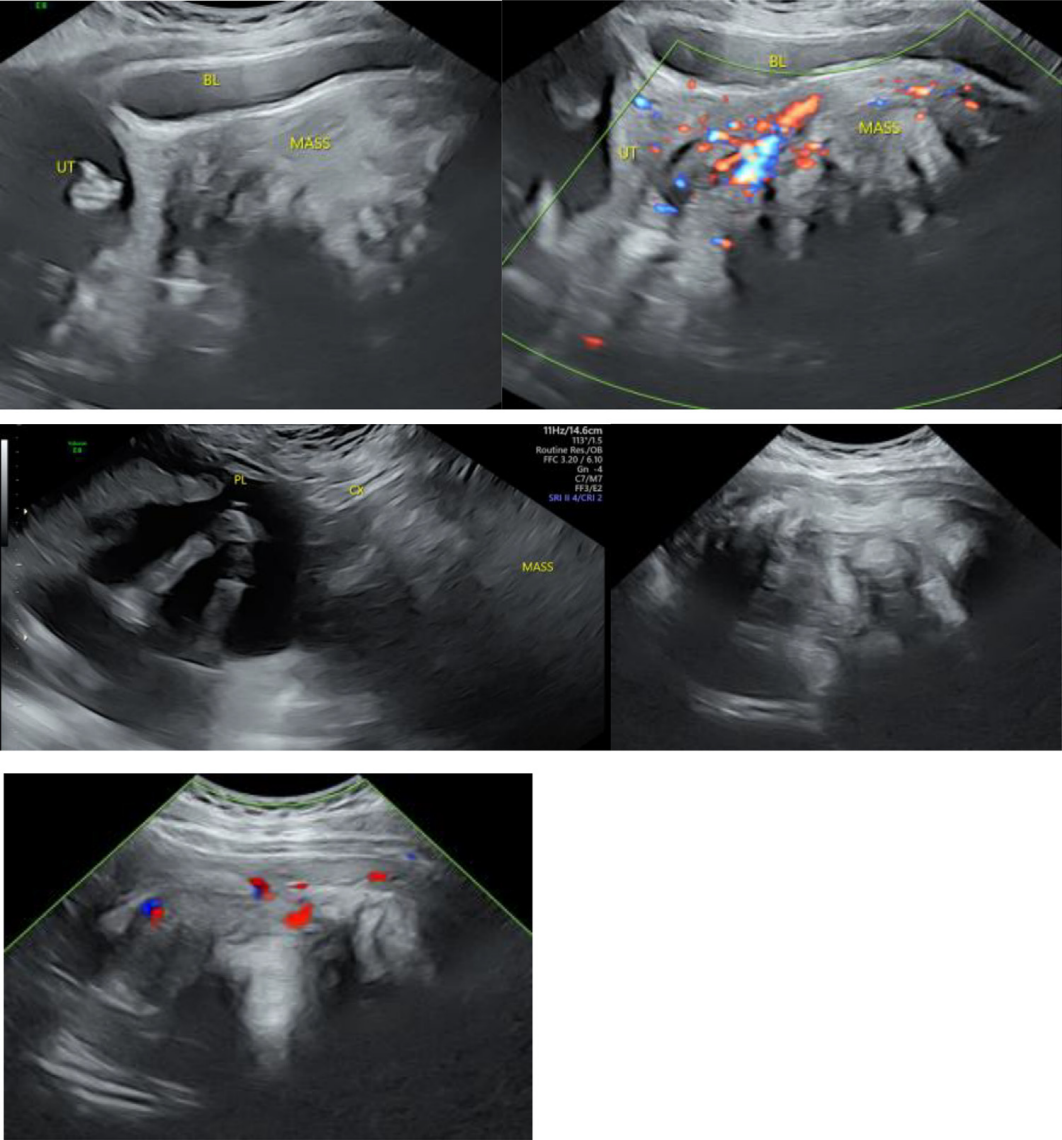
**Transperineal grayscale and color Doppler ultrasound images of the pelvis** showed a large, heterogeneous mass with posterior acoustic shadowing and significant vascularity, which was later diagnosed as AAM. It displaced the gravid uterus, cervix, and bladder superiorly. The mass could not be fully measured due to its massive size. Key: MASS = AAM, BL = bladder, UT = uterus, CX = cervix, PL = placenta.

Transabdominal ultrasound showed a large complex heterogeneous pelvic mass that filled the pelvis and could not be measured due to its large size, although a previous ultrasound from an outside facility reported a maximum dimension of 16 cm. Because a transvaginal approach led to patient discomfort, a transperineal ultrasound (Fig. 1) was performed and revealed that the mass was somewhat vascular and superiorly displaced the uterus and cervix. The right ovary was separate from this structure, and the left ovary was not seen.

**Fig. 2:**
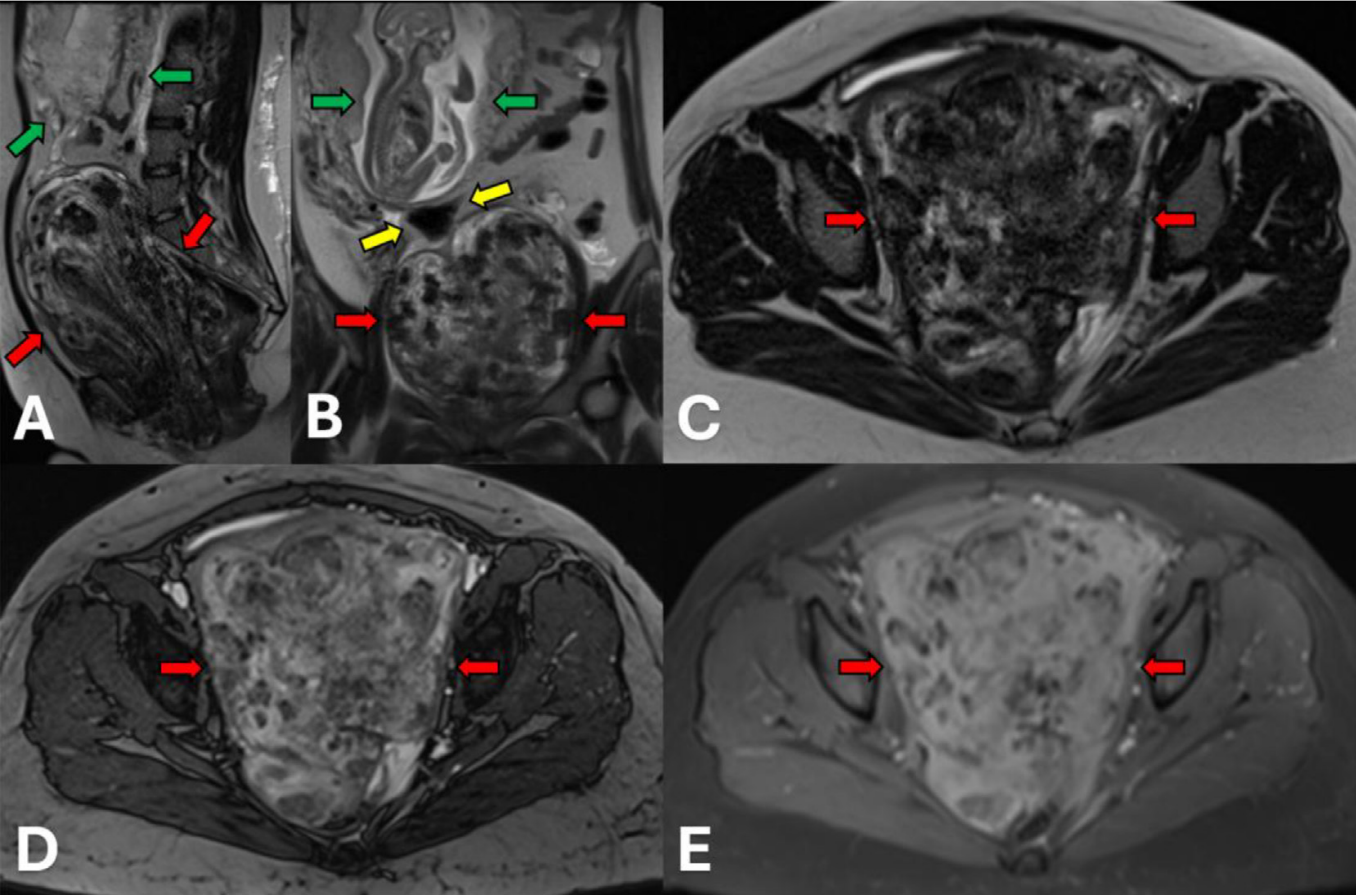
**Initial MRI of the pelvic AAM.** Sagittal (A), coronal (B), & axial (C) images from the half-Fourier acquisition single-shot turbo spin echo (HASTE) sequence demonstrated a 23 cm heterogeneous mass (biopsy-proven AAM, red arrows), which appears to arise from the lower cervix and vagina. Mass effect displaced the gravid uterus (green arrows) and bladder (yellow arrows) superiorly and to the right of midline. The ovaries (not pictured) were separate from the mass. Axial fat-suppressed image from the true fast imaging with steady-state free precession (TRUFI) pulse sequence (D) revealed the mass did not contain macroscopic fat. Axial T1-weighted image with fat suppression (E) showed several areas of punctate hyperintense signal indicative of hemorrhagic products and slow-flow vascular channels in the mass.

**Fig. 3:**
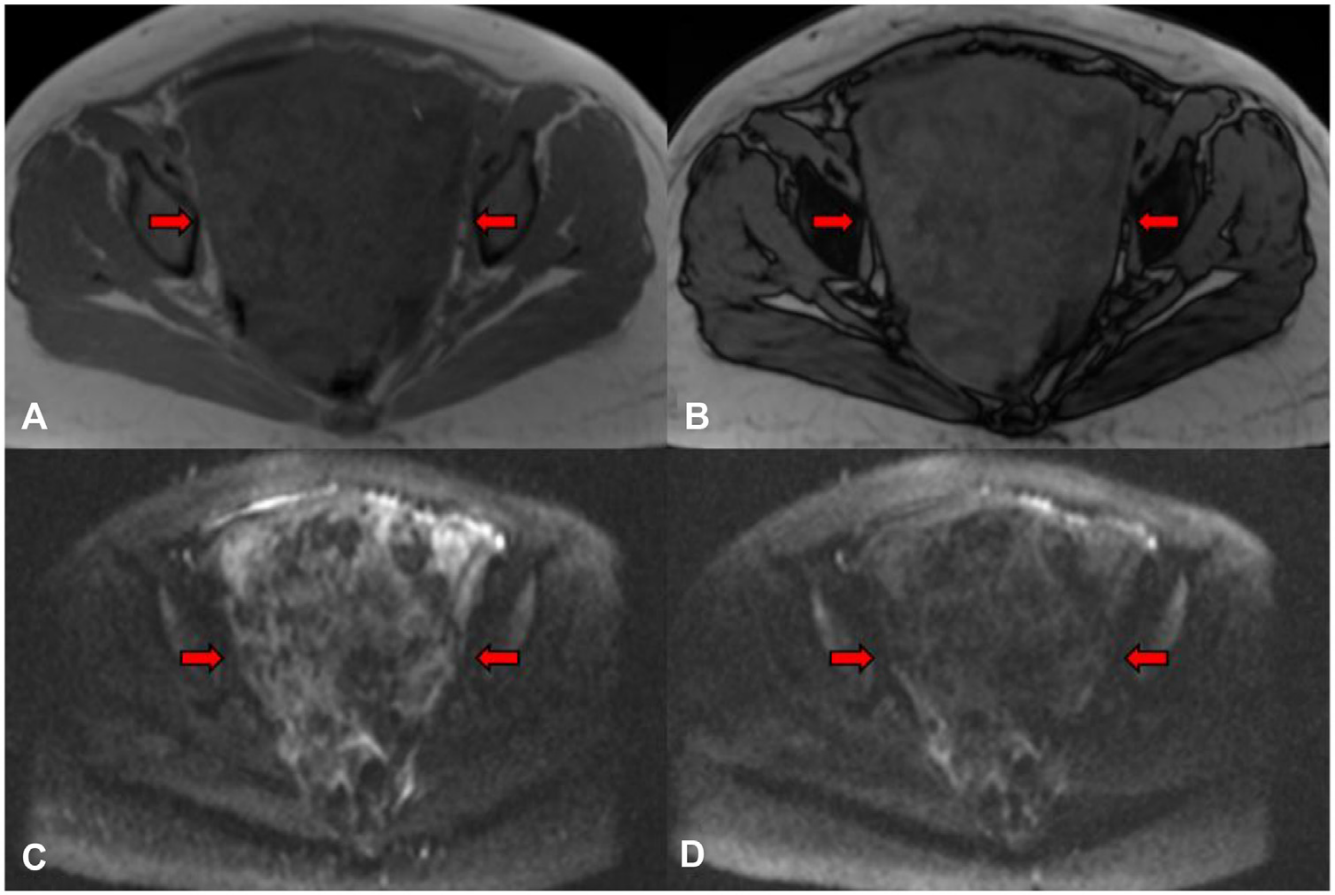
**Initial MRI of the pelvic AAM (continued).** Axial in-phase (A) and opposed-phase (B) images showed no significant change in signal between phases to suggest the presence of microscopic fat or iron deposition. Axial diffusion-weighted imaging (DWI) sequence (C) and apparent diffusion coefficient (ADC) map (D) revealed areas of mild diffusion restriction throughout the mass.

**Fig. 4:**
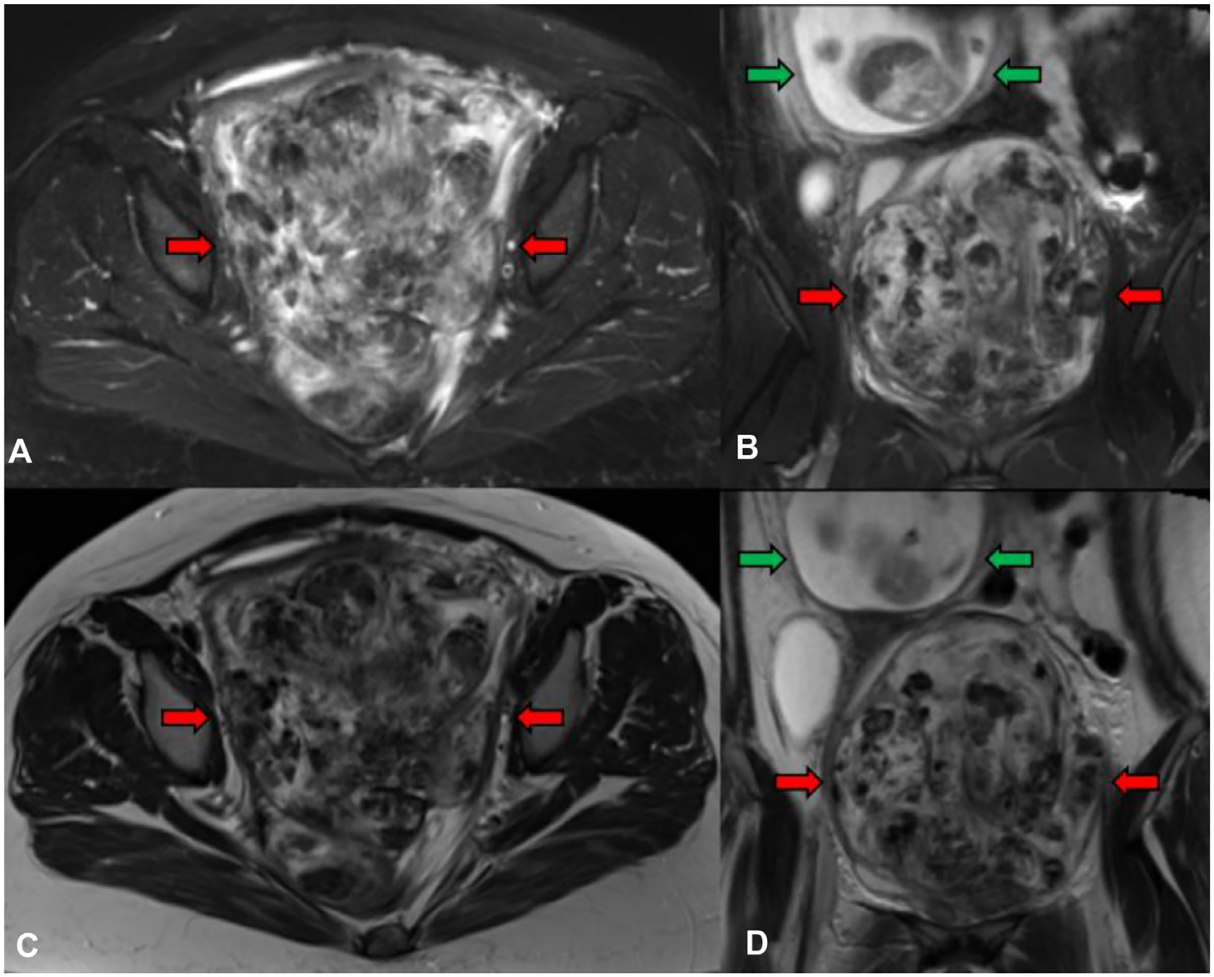
**Initial MRI of the pelvic AAM (continued).** Axial and coronal T2-weighted images demonstrated mixed areas of hypo- and hyperintense signals throughout the mass (red arrows). There was no significant signal dropout on the fat suppressed images (A, B) compared to the nonsuppressed images (C, D) to suggest the presence of macroscopic fat. The gravid uterus (green arrows) was displaced superiorly to the right of midline.

On the initial magnetic resonance imaging (MRI) of the abdomen and pelvis (Fig. 2, Fig. 3, Fig. 4), the mass measured 23.3 × 10.3 ×14.3 cm with punctate areas of T1 hyperintense signal, indicative of hemorrhagic products, along with T1 iso-hypointense and T2 hypo-hyperintense internal signal. It was lobulated, laminated, and heterogeneous and appeared to arise from the lower cervix/vagina. The mass insinuated between the internal and external sphincters on the left, and it traversed the ipsilateral pelvic diaphragm into the ischiorectal fossa, resulting in rightward displacement of the bladder.

**Fig. 5:**
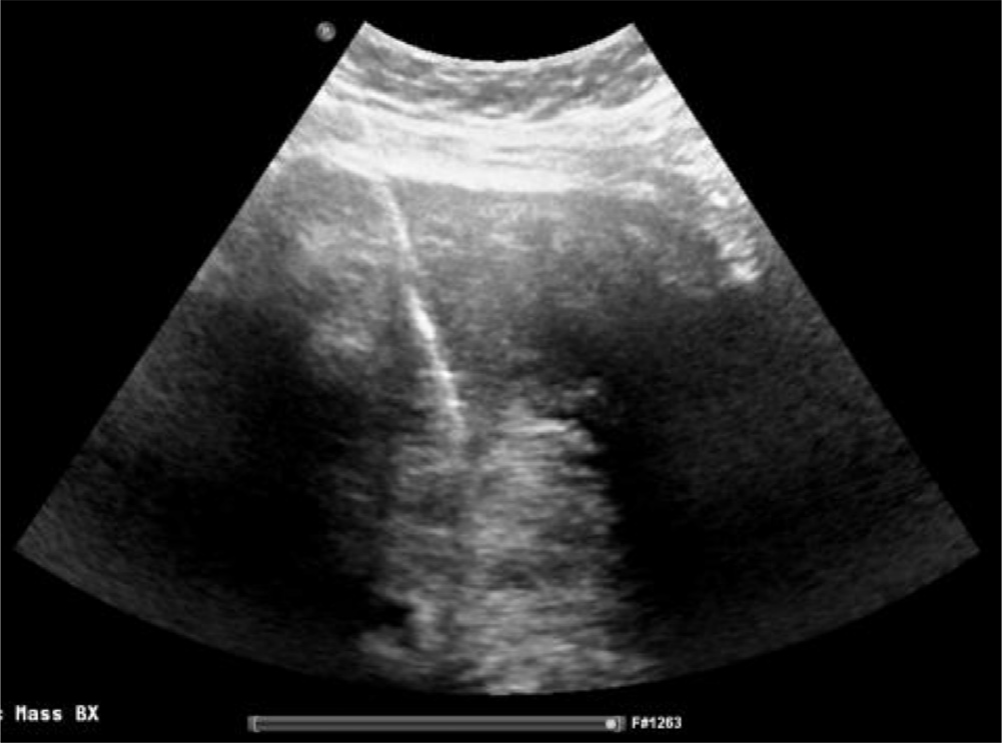
**Ultrasound-guided core needle biopsy** confirmed the position of the needle within the mass, which was diagnosed as AAM.

The patient was then referred to interventional radiology for ultrasound-guided percutaneous biopsy (Fig. 5), which was performed without complication. Histological sections showed a hypocellular neoplasm with myxoid matrix. Medium-to-large-sized vessels and smooth muscle bundles were present. Immunohistochemical stains were positive for estrogen receptors, progesterone receptors, and alpha smooth muscle actin but negative for desmin. The findings were consistent with a deep (aggressive) angiomyxoma.

**Fig. 6:**
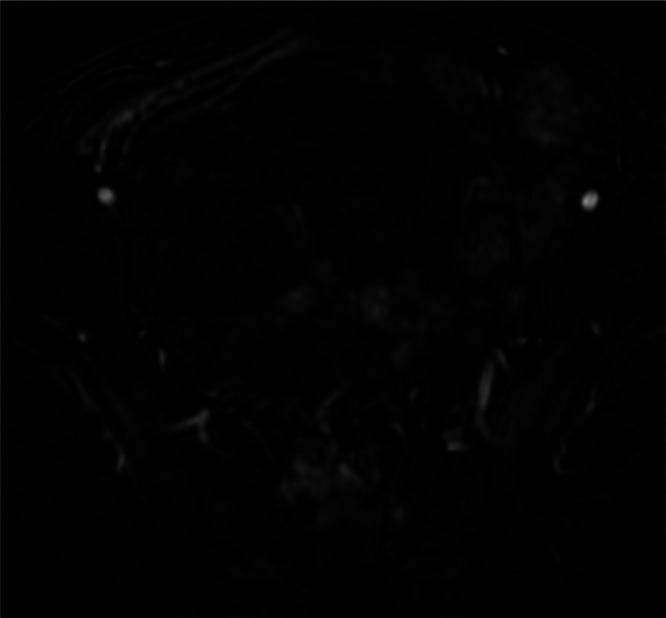
**Subtracted image from a 10-month follow-up contrast-enhanced pelvic MRI** at the level of the uterine arteries demonstrated the hypovascular nature of the AAM, which did not significantly reduce in size despite leuprolide therapy.

Unenhanced 1-month and 2-month follow-up MRIs of the pelvis demonstrated progressive enlargement of the mass up to 25.5 × 16.7 × 15.4 cm. Approximately 3 weeks later, the patient underwent classical cesarean section delivery at 34 weeks 0 days gestation and was established with gynecologic oncology for surveillance on leuprolide therapy at 3-month intervals. Follow-up contrast-enhanced MRIs of the pelvis revealed the mass to be relatively hypovascular with gradual shrinkage down to 21.5 × 12.1 × 11.5 cm nearly 9 months after the first leuprolide injection (Fig. 6). However, the patient reported increasing pelvic pain while on tramadol, so she was referred to interventional radiology for embolization of the mass to improve her symptoms and decrease surgical morbidity for potential resection.

**Fig. 7:**
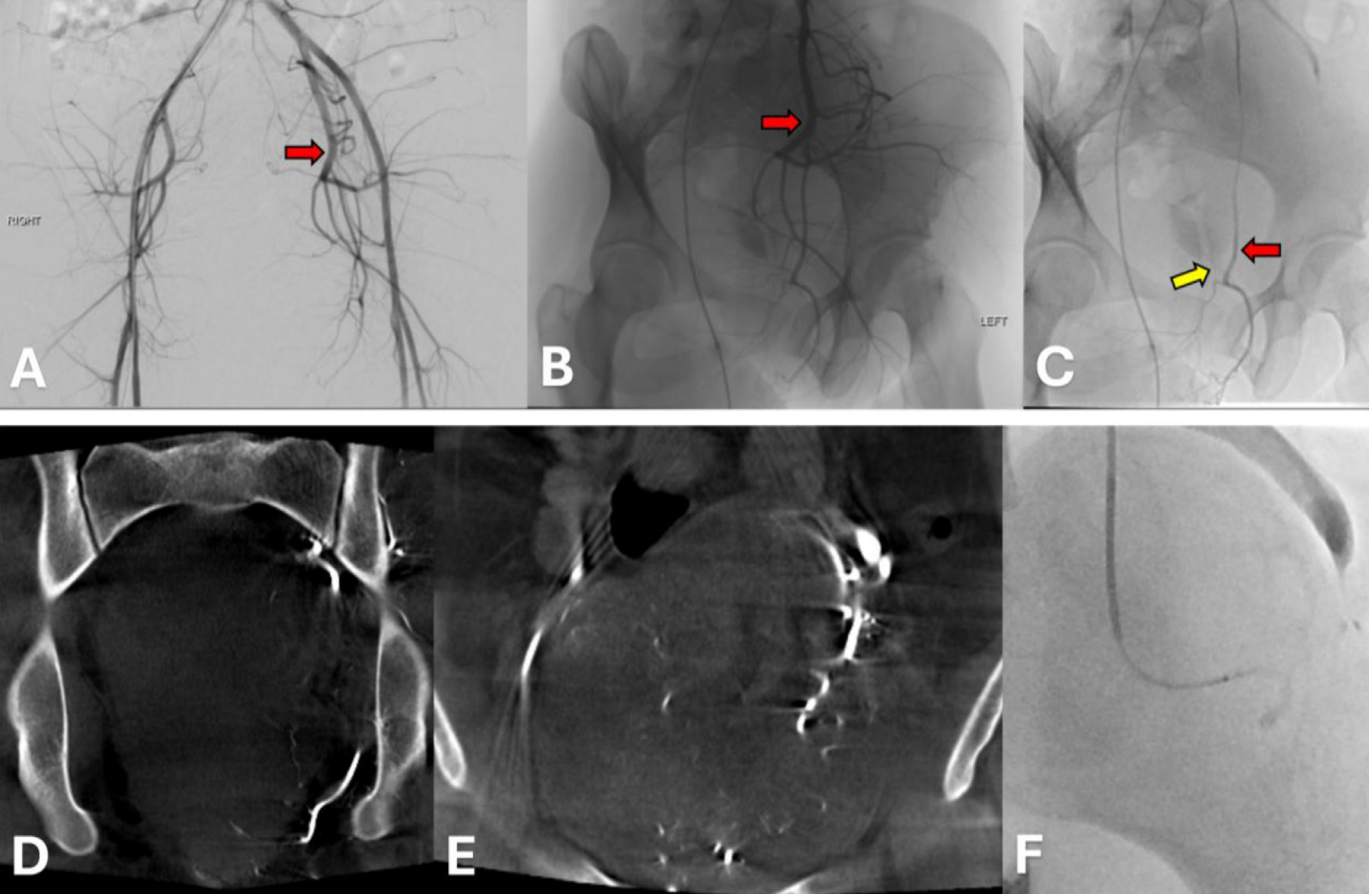
**Pelvic angiography and cone beam CTs with embolization of a hypovascular AAM through the left uterine artery. A:** A 5 Fr Contra flush catheter was advanced into the upper abdominal aorta (not pictured) via the right common femoral artery. Nonselective pelvic angiography in the AP projection revealed the mass had a dominant arterial supply from the left internal iliac artery (red arrow). **B:** A 5 Fr RBT catheter was used to select the left internal iliac artery (red arrow). Selective angiography in the right anterior oblique projection redemonstrated the findings in A. **C:** A 2.8 Fr Progreat microcatheter was advanced into the left uterine artery (red arrow). Angiography confirmed that the mass received most of its blood supply from this location. However, the mass itself was relatively hypovascular, similar to the contrast-enhanced pelvic MRI. Minimal arterial supply was seen in the bladder. **D & E:** Coronal intraoperative cone beam CTs confirmed a few distal branches of the left uterine artery predominantly supplied the mass. Incidentally, the patient was found to have two left ureters and one right ureter. **F:** Embolization of the left uterine artery was performed with 500-700 micrometer Embospheres. Post-embolization angiography revealed stasis of arterial flow to the mass.

Nonselective pelvic angiogram (Fig. 7) revealed a dominant arterial supply to the mass from the left internal iliac artery. The left internal pudendal artery provided a minimal supply, while the left uterine artery contributed multiple feeding vessels. The left uterine artery was thus embolized with 500-700 micrometer Embospheres, which resulted in near stasis. Two months later, the patient reported worsened pelvic pain at her follow-up gynecologic oncology appointment. While this was felt not to be uncommon after embolization, the increase in bulk symptoms related to mass growth was thought to be a contributing factor. However, an MRI of the pelvis performed the next day did not show any significant change in the size of the mass when compared to the MRI obtained 3 months prior to embolization.

**Fig. 8:**
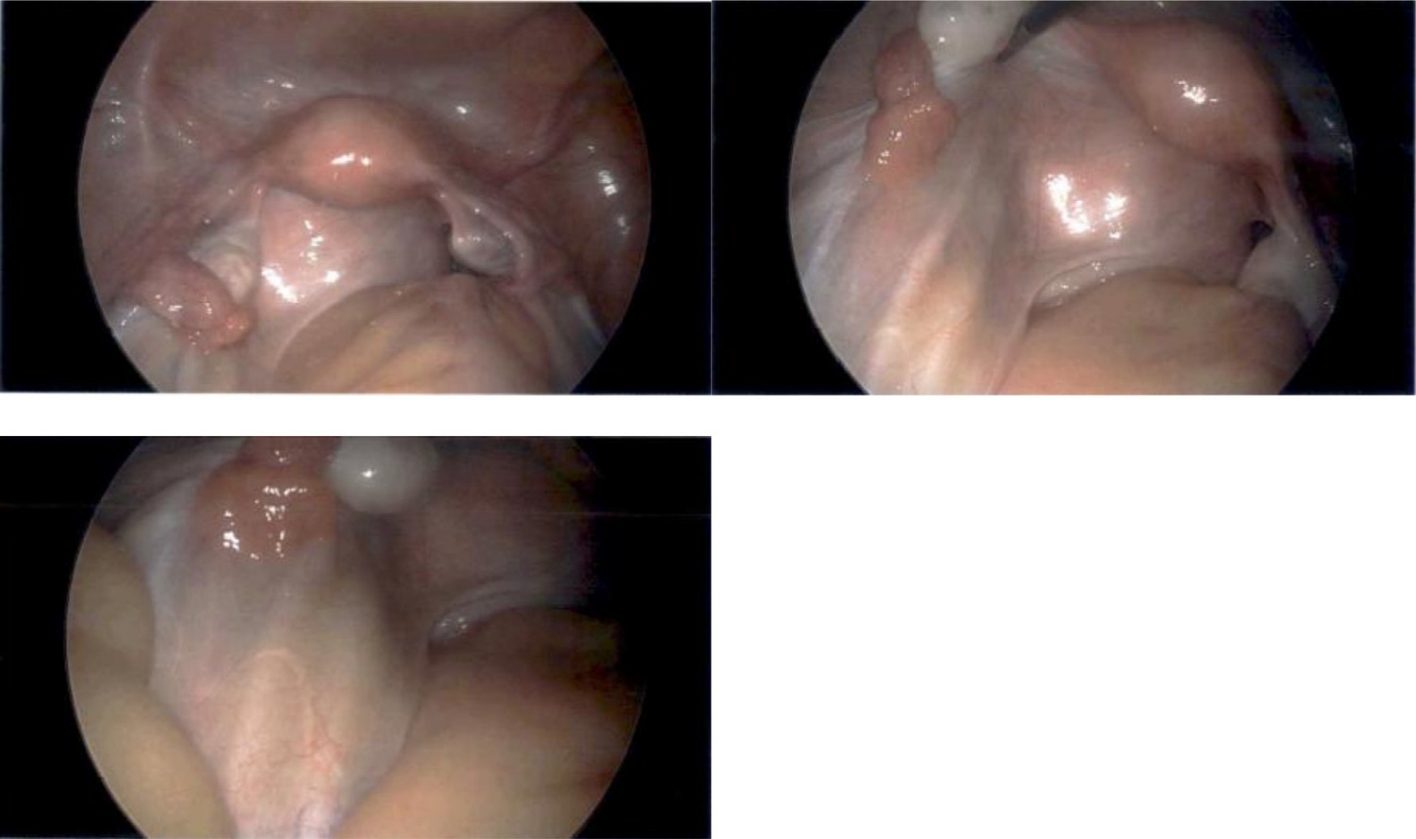
**Diagnostic laparoscopy** showed a mobile, retroperitoneal mass at the posterior and inferior aspect of the vagina and cervix without sidewall involvement. Flimsy adhesions were seen at the site of prior midline vertical incision at the level of the umbilicus. Incidentally, there were two ureters on the left and one ureter on the right, all with peristalsis.

After further discussion with gynecologic oncology, the patient agreed to proceed with tumor resection. The following month, the patient underwent diagnostic laparoscopy (Fig. 8), proctoscopy, and cystoscopy for preoperative planning. A mobile mass with dense adhesions was noted to be entirely at the posterior and inferior aspect of the vagina, cervix, and retroperitoneum with no obvious sidewall involvement. It exerted mass effect but did not invade the rectum, bladder, or ureters. As a result, the patient was deemed a good surgical candidate.

**Fig. 9:**
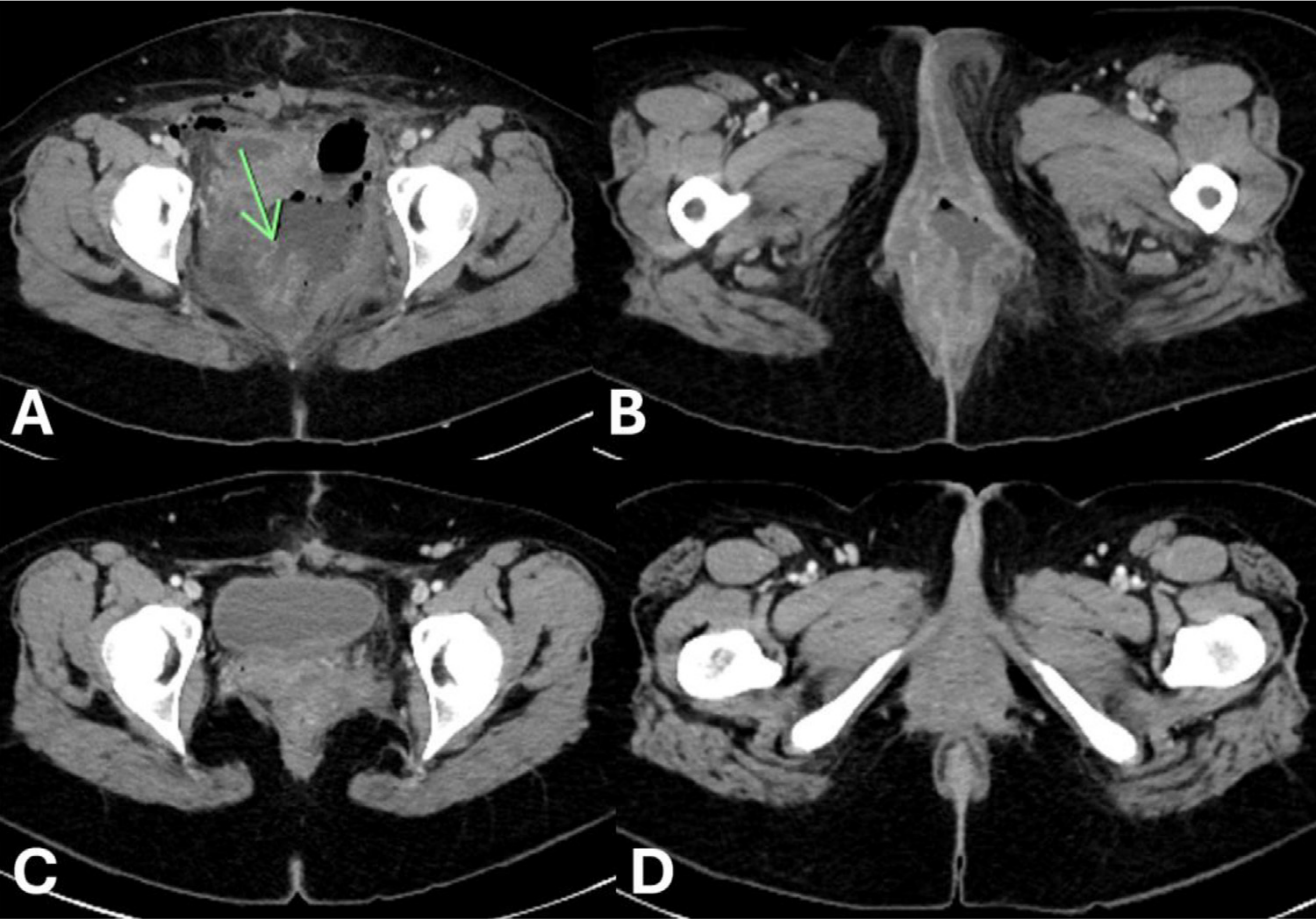
**Contrast-enhanced CT of the abdomen and pelvis performed after resection of the AAM** showed a pelvic fluid collection with air secondary to iatrogenic rectal injury (A). The procedure was also complicated by a rectovaginal fistula (green arrow) that allowed feculent material to enter the vaginal canal (B). The most recent follow-up CT scan (C, D) demonstrated resolution of these findings 42 days after resection.

Mass resection was performed 2 months later without significant intraoperative bleeding. However, the procedure was complicated by a rectovaginal fistula and a left perineal infection, which were confirmed by postoperative CT of the abdomen and pelvis (Fig. 9). The patient then underwent successful diverting loop colostomy creation, pelvic washout, and placement of two Penrose drains into the fluid collection, which progressively resolved on serial follow-up CT examinations up to 42 days after resection (Fig. 9). She was advised to follow up with gynecology and colorectal surgery for continued management. Serial pelvic examinations revealed a well-healed vagina without residual defects. Colorectal surgery recommended an additional CT and MRI as well as an endoscopy of the colostomy’s proximal and distal ends to determine her candidacy for colostomy reversal and closure. Of note, these studies were not yet obtained at the time this case report was accepted for publication.

## Discussion

AAMs are best evaluated on MRI, which typically shows a characteristic laminated internal architecture consisting of alternating T2 hyper- and hypointense layers, as seen in our patient. Marked contrast enhancement of the tumor is also expected on MRI due to its typical hypervascular properties [3], although our patient’s tumor was unusually hypovascular. Interestingly, the more benign superficial (cutaneous) angiomyxoma and intramuscular myxoma have variable degrees of vascularity, with many of them noted to be hypovascular [4,5]. In pregnant patients, ultrasound is particularly useful in the initial evaluation of AAMs when considering the risk of radiation exposure to the developing fetus. Findings on ultrasound commonly include a hypoechoic solid mass with cystic components in addition to increased internal and peripheral vascularity seen on color Doppler [6]. Unfortunately, ultrasound is rather limited in evaluating the true size of the mass, especially in larger AAMs, due to its limited field of view and, in some cases, posterior acoustic shadowing.

### Differential diagnosis

AAM can resemble multiple pelvic disease entities on medical imaging. Moreover, a hypovascular mass does not necessarily exclude AAM from the differential diagnosis. Below are some important examples along with their clinical and imaging properties that can help medical providers distinguish them from AAM.

### Bartholinitis

Labial swelling due to Bartholin gland inflammation can be clinically indistinguishable from AAM [7]. On MRI, a thick-walled, cystic mass with rim enhancement and infiltrative changes in adjacent fat is suggestive of inflammation rather than AAM [7]. However, Bartholin gland hyperplasia can have extensive mucin extravasation that mimics AAM [7].

### Myxoid smooth muscle tumors

Like AAM, these tumors may occur primarily in the vulva with circumscribed margins and T2-hyperintense signal on MRI [7]. Their smooth muscle composition enables them to arise from other pelvic structures like the perineum, uterus, and rectum [[8], [9], [10]], which can make them even more challenging to distinguish from AAM. Clinical history, in this case, may be useful since myxoid smooth muscle tumors of the female genital tract typically occur in the fourth to fifth decade of life [11], whereas AAM can present as early as the second decade [7].

### Vulval angiomyofibroblastoma

While morphologically similar to AAM, this neoplasm usually involves superficial soft tissues and tends to be smaller (< 5 cm) and more circumscribed [7]. AAM, on the other hand, often involves deeper structures within the pelvis and can grow larger than 5 cm [7].

### Mucinous soft tissue sarcomas

The higher T2 signal seen on MRI may mimic AAM [7]. However, these sarcomas typically lack the characteristic swirling pattern suggestive of AAM, which facilitates their diagnosis [7].

### Vulval carcinoma

This neoplasm has intermediate to high T2 signal intensity on MRI rather than the very high T2 signal intensity often seen with AAM [7]. Other features that set it apart from AAM include absence of the characteristic swirling pattern as well as its tendency to invade adjacent structures and metastasize to regional lymph nodes [7].

### Diagnostic checklist

Consider AAM in a premenopausal female with a large vulval mass that straddles the levator ani muscle [7]. On MRI, look for a large, T2-hyperintense perineal/pelvic mass with internal swirling/laminated pattern, which commonly straddles levator ani and displaces, rather than invades, adjacent structures [7].

### Clinical treatment

The management of AAM during and after pregnancy can be an especially challenging process for adolescent patients to navigate given their age and guardianship implications. Multidisciplinary counseling with obstetrics and gynecologic oncology is beneficial for patients to learn about the nature of their disease as well as the risks and benefits of the treatment options available to them. Inclusion of an ethics specialist can also inform adolescent patients of the complex dilemmas that may arise with respect to the possible complications during their pregnancy, the shift in responsibilities they could have as parents while undergoing postpartum treatment, and the potential alterations in their fertility for family planning.

Very large pelvic AAMs identified during pregnancy are a relative contraindication to surgical resection due to their local infiltration of sensitive organs, namely the gravid uterus, cervix, and vagina. Significant involvement of the latter also renders vaginal delivery difficult given the mechanically obstructive effects from the tumor. Fortunately, our patient was able to deliver via classical cesarean section without any adverse, iatrogenic outcomes related to the AAM. A hysterectomy was also not indicated given the tumor's relatively mild uterine infiltration and the patient’s desire to remain fertile.

In the postpartum period, our patient was offered surgical versus medical management of the neoplasm. Surgical resection is the recommended treatment but is often difficult due to the bland myxoid histologic pattern, which blends into the surrounding tissue. Adequate margins are therefore difficult to achieve and local recurrence is high. Neoadjuvant therapies such as hormonal treatments and embolization are proposed to decrease tumor burden. In our patient, leuprolide therapy was moderately successful, resulting in a 54.4% reduction in tumor volume (using ellipsoid approximation) despite her symptomatic progression. Embolization can be performed to devascularize the tumor and induce ischemia. However, the presence of multiple arteries providing vascularization to the tumor may cause a varied level of response to treatment. Additionally, our patient’s mass was relatively hypovascular, which is uncommon for AAMs and likely explains its poor response to embolization. In the case of future resection, the subsequent devascularization and ischemia following embolization can make the tumor less prone to intraoperative bleeding and easier to distinguish from the surrounding tissue, thus facilitating resection [12,13]. Fortunately, our patient was able to undergo diagnostic laparoscopy, proctoscopy, and cystoscopy without complication to confirm the mass did not invade sensitive structures like the rectum, bladder, and ureters. Additionally, the patient did not have large-volume active bleeding during mass resection, which was performed 5 months after embolization.

## Conclusion

The proliferative and occasionally invasive nature of AAM makes imaging a vital tool to localize its involvement of adjacent structures for surgical planning. While ultrasound is an appropriate screening modality to evaluate pelvic masses discovered on gynecologic exams, MRI is the preferred modality to assess the entire scope of not only what the mass represents but also how its mass effect, possible invasion, and degree of vascularity affect whether a patient is a good candidate for targeted embolization and resection, especially if the mass does not respond well to medical management. Targeted embolization can serve to induce ischemia, which often better delineates the mass’s boundaries from the surrounding tissues; however, tumor shrinkage and symptomatic relief do not consistently occur due to the variable vascular composition and stimulating hormonal influences associated with AAMs. Finally, other surgical approaches like laparoscopy, proctoscopy, and cystoscopy can confirm whether mass invasion is present.

## Patient consent

Written informed consent has been obtained from the patient for publication of this case report and accompanying images.
